# Clinical Evaluation of the Measurement Performance of the Philips Health Watch: A Within-Person Comparative Study

**DOI:** 10.2196/mhealth.6893

**Published:** 2017-02-02

**Authors:** Jos Hendrikx, Loes S Ruijs, Lieke GE Cox, Paul MC Lemmens, Erik GP Schuijers, Annelies HC Goris

**Affiliations:** ^1^ Innovation Site Eindhoven Philips Eindhoven Netherlands; ^2^ Philips Research Eindhoven Netherlands

**Keywords:** sedentary lifestyle, monitoring, ambulatory, monitoring, physiologic, accelerometry, actigraphy, photoplethysmography, heart rate, energy metabolism, adult, humans

## Abstract

**Background:**

Physical inactivity is an important modifiable risk factor for chronic diseases. A new wrist-worn heart rate and activity monitor has been developed for unobtrusive data collection to aid prevention and management of lifestyle-related chronic diseases by means of behavioral change programs.

**Objective:**

The objective of the study was to evaluate the performance of total energy expenditure and resting heart rate measures of the Philips health watch. Secondary objectives included the assessment of accuracy of other output parameters of the monitor: heart rate, respiration rate at rest, step count, and activity type recognition.

**Methods:**

A within-person comparative study was performed to assess the performance of the health watch against (medical) reference measures. Participants executed a protocol including 15 minutes of rest and various activities of daily life. A two one-sided tests approach was adopted for testing equivalence. In addition, error metrics such as mean error and mean absolute percentage error (MAPE) were calculated.

**Results:**

A total of 29 participants (14 males; mean age 41.2, SD 14.4, years; mean weight 77.2, SD 10.2, kg; mean height 1.8, SD 0.1, m; mean body mass index 25.1, SD 3.1, kg/m^2^) completed the 81-minute protocol. Their mean resting heart rate in beats per minute (bpm) was 64 (SD 7.3). With a mean error of −10 (SD 38.9) kcal and a MAPE of 10% (SD 8.7%), total energy expenditure estimation of the health watch was found to be within the 15% predefined equivalence margin in reference to a portable indirect calorimeter. Resting heart rate determined during a 15-minute rest protocol was found to be within a 10% equivalence margin in reference to a wearable electrocardiogram (ECG) monitor, with a mean deviation of 0 bpm and a maximum deviation of 3 bpm. Heart rate was within 10 bpm and 10% of the ECG monitor reference for 93% of the duration of the protocol. Step count estimates were on average 21 counts lower than a waist-mounted step counter over all walking activities combined, with a MAPE of 3.5% (SD 2.4%). Resting respiration rate was on average 0.7 (SD 1.1) breaths per minute lower than the reference measurement by the spirometer embedded in the indirect calorimeter during the 15-minute rest, resulting in a MAPE of 8.3% (SD 7.0%). Activity type recognition of walking, running, cycling, or other was overall 90% accurate in reference to the activities performed.

**Conclusions:**

The health watch can serve its medical purpose of measuring resting heart rate and total energy expenditure over time in an unobtrusive manner, thereby providing valuable data for the prevention and management of lifestyle-related chronic diseases.

**Trial Registration:**

Netherlands trial register NTR5552; http://www.trialregister.nl/trialreg/admin/rctview.asp?TC=5552 (Archived by WebCite at http://www.webcitation.org/6neYJgysl)

## Introduction

With the increase in passive transportation, spectator-based entertainment, and decreases in energy expenditure through decreased activity during occupational and household work, modern life has evolved to eliminate many forms of physical labor that were prevalent in earlier times [1,2]. Together with the rise in sedentary lifestyles, the incidence of chronic and noncommunicable diseases (NCDs) such as cardiovascular diseases, cancers, chronic respiratory diseases, and diabetes has risen. According to the World Health Organization (WHO), NCDs kill 38 million people each year [3]. Of those NCD deaths, 16 million occur “prematurely” before the age of 70 years. With 17.5 million per year, cardiovascular diseases account for most NCD deaths, followed by cancers (8.2 million), respiratory diseases (4 million), and diabetes (1.5 million). Modifiable behavioral risk factors for NCD development include tobacco and alcohol use, unhealthy diet, and insufficient physical activity [3]. Dietary risk factors and physical inactivity collectively accounted for 10% of global disability-adjusted life years in 2010 [4]. Additionally, approximately 3.2 million deaths annually can be attributed to insufficient physical activity, making this a primary candidate for low-cost interventions aimed at preventing and controlling the impact of NCDs. Guideline and recommendation documents based upon extensive literature reviews from the WHO, European Society of Cardiology, American College of Sports Medicine (ACSM), American Heart Association, American College of Cardiology, American Diabetes Association, and US Preventive Services Task Force all come to the same conclusion regarding physical activity and health: a sufficient level of physical activity is key in primary and secondary prevention of chronic lifestyle-related diseases [5-19]. For adults, at least 150 minutes of moderate to vigorous physical activity in bouts of at least 10 minutes in duration per week is generally recommended by the aforementioned organizations [6,9-14,16,17,20,21].

To help people act on physical activity recommendations, accurate assessment of the intensity, for example, expressed as energy expenditure, and duration of their physical activity is required. Currently, a considerable number of devices are on the market that enable this assessment by their users. These devices predominantly operate using accelerometers to estimate energy expenditure. Recently, technology has advanced, enabling inclusion of a photoplethysmography (PPG) sensor in these devices for the measurement of heart rate. Data from this additional sensor can enable more accurate estimations of energy expenditure because heart rate has been shown to have a linear relationship with oxygen consumption (a measure for energy expenditure) during moderate- and high-intensity activity [22-24]. Using PPG to derive heart rate from the blood volume pulse observed in the microvascular tissue [25], by exploiting the inverse relationship between blood volume and amount of light reflected, has been used for decades in clinical applications such as pulse oximetry and vascular diagnostic tools [26]. The accuracy of the PPG-derived heart rate estimation compared with electrocardiogram (ECG)-based reference measurements has been validated as highly reliable [26-29]. However, when considering energy expenditure, more variable performance of the multisensor technology devices has been observed [27,30]. For instance, Lee et al [30] observed a mean absolute percentage error (MAPE) of 23.5% for the Basis B1 multisensor device.

Another device exploiting multisensor technology for energy expenditure estimation is the Philips health watch, which makes use of the Philips Cardio and Motion Monitoring Module (CM3-Generation-3), an accelerometer as well as a PPG sensor module developed by the Philips Wearable Sensing Technologies (WeST) division. Its purpose is seamless daily monitoring of heart rate and physical activity and deriving clinically relevant parameters such as total energy expenditure, resting heart rate, step count, and types of activity performed. Tracking these parameters enables self-care and (automated) coaching in health plans complying with aforementioned guidelines, to minimize the risk of developing chronic lifestyle-related diseases and to manage existing morbidity. Monitoring of physical activity as done with the Philips health watch can give users important feedback regarding their daily status on overall activity level, including activity intensity and duration.

Next to heart rate and total energy expenditure, resting heart rate is an important clinical parameter, which the Philips health watch estimates as well. Systematic reviews and meta-analyses indicate that high resting heart rate is an important risk factor for adverse health outcomes, including all-cause mortality, cardiovascular mortality, cardiovascular diseases, and type 2 diabetes [31-35]. In addition to being an informative risk factor, resting heart rate has been demonstrated to be a modifiable treatment outcome [36-47]. Although drug therapy has been shown to result in the largest decline in elevated resting heart rate, exercise therapy has also been shown to reduce resting heart rate [41-44]. Measuring and monitoring resting heart rate, and preventing long-term increases in an individual’s resting heart rate by suitable exercise therapy, can therefore support reduction of the risk of adverse health outcomes. In clinical context, resting heart rate is generally measured by asking a person to sit or lie down for 5-15 minutes, during which the heart rate is measured. The heart rate after a short settling period is then considered to be representative of the resting heart rate [48-50]. It should be noted that this resting heart rate measurement is influenced by the duration of the resting period before taking the measurement, posture, and environmental conditions [50,51]. The Philips health watch derives resting heart rate values automatically from continuous heart rate measurements throughout the day, applying automatic selection of periods where a user is in a resting state but not asleep.

The aim of this study was to evaluate performance of total energy expenditure and resting heart rate measures of the health watch. Secondary objectives were to assess the performance of the Philips health watch with respect to its continuous measurement of heart rate, the estimation of respiration rate at rest and the number of steps a user takes, and the correct classification of activity types: walking, cycling, running, and other.

## Methods

### Study Design and Compliance

The study was designed as a within-person comparative study where parameters estimated by the Philips health watch (DL8791, Philips, Stamford, CT, USA) were compared with measurements of reference devices. The study was performed in compliance with ISO (International Organization for Standardization) 14155 “Clinical investigation of medical devices for human subjects – Good clinical practice,” the Declaration of Helsinki, and local regulations. An independent medical ethics committee (METC Brabant) approved the study and it was registered in the Netherlands Trial Registry (NTR5552). Before participation all subjects gave written informed consent.

### Objectives

The primary objective of this study was to determine, in a clinical study, the accuracy of the Philips health watch regarding the estimation of total energy expenditure and resting heart rate. Secondary objectives included the assessment of accuracy of other output parameters of the monitor: heart rate, step count, activity type, and respiration rate at rest.

### Study Population

For this study, adult (≥18 years) participants with a body mass index between 19 and 35 kg/m^2^ were recruited from the Dutch general population. Respondents with any of the following criteria were excluded from participation in the study: pregnancy, presence of skin conditions or wounds in the wrist area, presence of a chronic disease for which a physician had contraindicated moderate-intensity exercise without medical supervision, presence of a pacemaker or other implantable electronic device, or presence of a functional or cognitive impairment preventing compliance with the study protocol.

### Clinical Procedures

For each participant, the study started with an evaluation of his or her eligibility based on the inclusion and exclusion criteria and, if positive, giving informed consent. Then participants completed the Fitzpatrick skin type questionnaire [52] and received detailed information about the study procedure and the health watch that they received. Subsequently, a participant wore the health watch at home for 3 days during which he or she could carry out his or her normal daily life activities to gather free-living data; participants did not wear reference devices during the free-living period. The participants then performed a laboratory test comprising a variety of daily life activities, during which data were collected using both the health watch and various reference devices. The purpose of this laboratory test was to assess the accuracy of multiple health watch parameters compared with reference measurements.

The standardized laboratory protocol comprised the following activities: (1) indoor activities including rest (watching television) for 15 minutes and treadmill (4.5 km/h), treadmill uphill 5% (3 km/h), ergometer bike (60 rpm), cross trainer (60 W), household activities (mixture), desk work, lying down, and standing for 3 minutes each and (2) outdoor activities including walking, cycling, and running for 3 minutes each.

After each activity, there was at least 3 minutes of rest. The 15 minutes of rest at the start of the protocol was included for measuring resting heart rate in the laboratory and respiration rate at rest. The data from this activity were included in the cumulative energy expenditure that was analyzed for the primary objective. During this period, participants were sitting on a chair while watching an emotionally neutral documentary on a television. The mixture of household activities consisted of three 1-minute subactivities: washing dishes, folding towels and handkerchiefs, and vacuum cleaning.

If the outside temperature was less than 10°C (to remain compliant with the K4b^2^ instructions for use) or when it was raining, the outdoor activities were performed indoors. That is, participants walked in the corridors at their own pace, cycled at their own pace on the ergometer bike, and ran on the treadmill at a pace that they set themselves. This occurred for 8 participants. Participants were asked to eat a light breakfast or lunch before the test, to not take caffeine or smoke in the 2 hours before their appointment, and to not carry out intense physical activity in the period before the test.

### Investigational Device and Comparators

**Figure 1:**
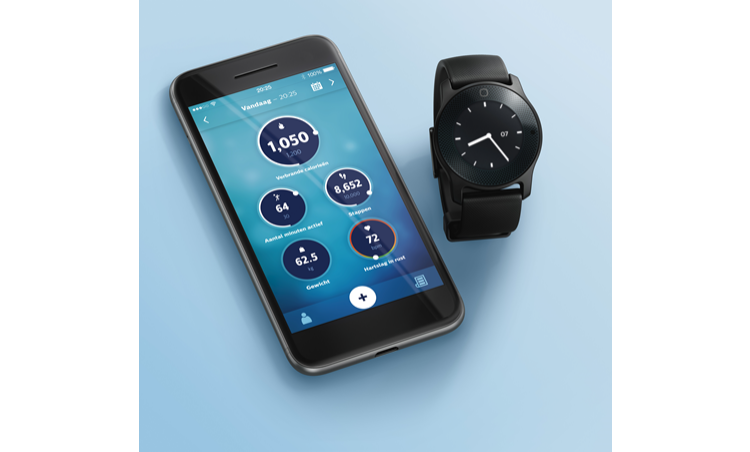
The Philips health watch with companion app running on a mobile phone.

The investigational device for this study was the Philips health watch, a wrist-worn, PPG-based, heart rate and activity monitor (Figure 1). The watch measures the health parameters at a 1-Hz sampling rate and displays real-time heart rate values and daily cumulative values for steps, active energy expenditure, and total energy expenditure. The 1-minute average values for heart rate, and cumulative steps and energy expenditure over 1 minute, are logged in internal memory and transmitted via Bluetooth to a phone running the companion app for 24/7 monitoring. The companion app displays the parameters over time to provide insights to the user by, for instance, color coding optimal or suboptimal parameter values based on personalized settings that are automatically determined from international standards (i.a. WHO and ACSM) and based on user input and input from the health watch. Additionally, based on a user’s personal program for achieving, for instance, a certain daily energy expenditure, coaching cards pushed via the app provide further insight and motivation to promote behavior change toward a healthier lifestyle.

#### Total Energy Expenditure

For total energy expenditure, the medical reference instrument was a K4b^2^ (COSMED, Rome, Italy). The K4b^2^ is a portable gas analysis system that was designed to be worn during (sports) activities. This instrument has been shown to be valid for the measurement of total energy expenditure [53,54]. For registering heart rate by the K4b^2^ device, participants wore a Polar T34 chest strap (Polar Electro Inc, Lake Success, NY, USA). For assessment of total energy expenditure estimation accuracy of the Philips health watch, cumulative total energy expenditure over the entire laboratory protocol as estimated by the Philips health watch was compared against cumulative total energy expenditure measured by the K4b^2^.

#### Resting Heart Rate

The Actiwave Cardio (CamNtech, Cambridge, UK) was the reference device for resting heart rate. It is a single-channel ECG waveform recorder that participants wore (only) during the laboratory protocol and it reported heart rate at a frequency of 1 Hz. Following current recommendations [50,51,55], resting heart rate was acquired from the 15-minute rest at the beginning of the laboratory protocol for the Philips health watch as well as the Actiwave Cardio because no reference measurements were taken during the free-living period. Resting heart rate was derived by taking the lowest 5-minute median heart rate value (determined using a sliding-window approach; that is, taking a subset of the data, with a length of 5 minutes, that stepped forward through the data at 1-second increments) during the rest period where participants were watching television for the Actiwave as well as the health watch. The lowest 5-minute median was chosen to derive resting heart rate values that were minimally influenced by measurement artifacts or disturbances of the resting condition of a participant.

In addition, the free-living heart rate and resting heart rate data were visually evaluated for all participants to verify that the automatic resting heart rate estimation of the health watch did indeed reflect participants’ heart rate in resting conditions.

### Heart Rate

For assessing the accuracy of heart rate, the health watch heart rate was evaluated over the whole duration of the laboratory protocol. For this purpose, based on the 1-Hz sampled values, mean heart rate values were calculated for 10-second nonoverlapping epochs for the duration of the laboratory protocol for both the reference device (Actiwave Cardio) and the Philips health watch. These data were then compared between devices to determine error values and coverage values, which were defined as the percentage of time the difference between both devices (either absolute in beats per minute, bpm, or relative in percentage compared with the reference) was within specific limits (10 bpm and 10%, respectively).

### Resting Respiration Rate

As a reference device for the respiration rate during rest, the K4b^2^ was used, which has been validated for this parameter [56]. The respiration rate was evaluated over the rest part of the laboratory protocol during which the participants were watching television. The mean respiration rate from the K4b^2^ during this activity was compared with the mean respiration rate of the Philips health watch.

### Step Counting

The accuracy of the step counting algorithm of the Philips health watch was determined by comparing with a Fitbit One (Fitbit Inc, San Francisco, CA, USA) device that was clipped onto participants’ trouser pockets as per manufacturer’s instruction. This waist-mounted step counter has been shown to be highly accurate for step counting during walking [57-59]. As a measure of the accuracy of the step counting algorithm for walking and running activities, the total number of steps for all these activities in the protocol was compared between the Philips health watch and the waist-mounted reference.

### Activity Type Recognition

Activity type recognition was compared against the (reference) list of activities from the protocol. The health watch classifies measurement data into 4 different types of activities (walking, running, cycling, and other), where changes between activity types are registered with a corresponding time stamp. Each activity from the laboratory protocol was timed using markers at the beginning and end of each activity that were set by the researcher using a Garmin Forerunner 620 (Garmin International Inc, Olathe, KS, USA). The activity type classifications for the laboratory activities were defined as follows: both treadmill exercises and outdoor walking were defined as walking, stationary cycling and outdoor cycling were defined as cycling, outdoor running was defined as running, and, except for the cross-trainer activity, the remaining activities were defined as other. The cross-trainer activity was not taken into account for determining the accuracy of health watch activity type recognition, as it could be classified as walking or running depending on the intensity at which the participant performed the task. The accuracy of activity type recognition was determined by calculating the average percentage of correct classifications of the consecutive activity type outputs of the device compared with the reference activity type on a second-by-second basis.

### Statistical Analysis

#### Sample Size Calculation

A 20% margin of equivalence has international consensus for the assessment of equivalence of medicinal products [60,61]. No such guidance exists for medical device comparisons; however, we chose to use more stringent margins in an effort to assess more meaningful equivalence to the reference measures while balancing the sample size of the study. Sample size was calculated for total energy expenditure equivalence compared with mobile metabolic measurements with a K4b^2^ system, based on a 15% margin of equivalence, and for resting heart rate compared with ECG measurements with an Actiwave Cardio device based on a 10% margin of equivalence. As the equivalence margins were expressed as percentages, the statistical hypotheses were expressed in terms of ratios instead of mean differences. Furthermore, data were log-transformed to enable conventional analysis in terms of a difference [62]. Subsequently, the sample size was calculated in Minitab version 17 (Minitab Inc) as the minimal number of participants needed to achieve a significance level of .05 and a power of .8 for the total energy expenditure objective as well as the resting heart rate objective for an equivalence test of paired means. For the sample size calculations, we used data from internal tests (see Table 1) with similar protocols to estimate the expected mean and SD of the (log-transformed) ratio between the health watch and reference measurements for both total energy expenditure and resting heart rate. Factoring in a 10% loss to follow-up, a total of 31 subjects were recruited.

**Table 1:** Means and standard deviations of the log-transformed ratio between the health watch measurements (x) and the reference measurements (y) based on data from internal tests that were used to determine the sample size for this trial.

Equation	Total energy expenditure	Resting heart rate
μ(log(*x*) – log(*y*))	0.925	1.032
σ(log(*x*) – log(*y*))	0.173	0.055

#### Analysis of Primary and Secondary Outcomes

For the primary outcomes, total energy expenditure and resting heart rate, equivalence tests of paired means were performed. As explained, log-transformation enabled conventional analysis in terms of a difference [62]. Using this transformation, the primary outcomes were tested using the two one-sided tests (TOST) approach for testing equivalence, applying paired sample *t* tests, at a significance level alpha of .05 and the predefined margins of equivalence [62]. In addition, 95% CIs of the difference of the means were determined, also expressed as ratio and calculated using log-transformation of the ratios.

For the secondary outcomes step count and resting respiration rate, equivalence tests for means were performed, similar to the primary outcomes. For both parameters, equivalence margins were set at ±10% compared with the reference measurement. Additionally, mean errors, mean absolute errors, mean percentage errors, and MAPEs were calculated. For activity type recognition, the accuracy was measured in the form of a confusion matrix, denoting the probability that the device classifies a certain activity, given a certain activity performed by the participants.

Before data analysis, all data were resampled to a common 1-Hz resolution. Data processing and analyses of primary and secondary outcomes were performed using MATLAB R2014b (The MathWorks, Inc).

#### Data Exclusion

Because of a history of epilepsy resulting in a safety hazard for laboratory testing in the trial, 2 subjects were excluded from participation. Another 2 participants (P107 and P114) experienced an adverse event that was classified as nonserious and not device-related after assessment by the trial’s independent medical monitor. Some data of participants were excluded from specific analyses because data were not correctly logged or, based on objective criteria, were found to be invalid (see Table 2).

These data exclusions led to the following numbers of participants available for each analysis: total energy expenditure, n=26; resting heart rate, n=23; heart rate, n=23; step counting, n=29 (overall); activity type recognition, n=26; resting respiration rate, n=28.

**Table 2:** Overview of participant data that were excluded entirely or partially from analysis.

Participant	Total energy expenditure	Resting heart rate	Heart rate	Respiration rate at rest	Step count	Activity recognition
P122^a^	X^b^	X	X	X	X	X
P127^a^	X	X	X	X	X	X
P107^c^	X	X	X	X		
P114^c^	X	X	X	X		
P102^d^	X			X		
P104^d,e^		X	X		X	X
P106^d^		X	X			
P128^d^		X	X			
P126^f^		X	X			

^a^Participant excluded before data collection.

^b^Crosses indicate for which analyses the data of the respective participant were deleted.

^c^Data excluded owing to possible influence of adverse event.

^d^Data excluded owing to reference device malfunction.

^e^Data excluded owing to incorrect execution of treadmill walking.

^f^Data excluded owing to heart rate being invalid (logged as 0).

## Results

### Participant Characteristics

A total of 31 participants were recruited, of whom 2 were excluded before data collection. This left 29 participants who took part in the trial, 14 male and 15 female. We observed the following distribution of the 6 Fitzpatrick skin types (1-6): n=0, 7, 18, 4, 0, 0. Demographics are presented in Table 3.

**Table 3:** Overview of participants’ (averaged) demographic characteristics.

Characteristic	Mean	Minimum	Maximum	SD
Age, years	41.2	18	65	14.4
Weight, kg	77.2	60	102	10.2
Height, m	1.8	1.6	1.9	0.1
BMI^a^, kg/m^2^	25.1	20.4	31.5	3.1
RHR^b^, beats per minute	64	49	77	7.3
BMR^c^, kcal/day	1654	1361	2082	216.7

^a^BMI: body mass index.

^b^RHR: resting heart rate.

^c^BMR: basal metabolic rate.

### Total Energy Expenditure

**Figure 2:**
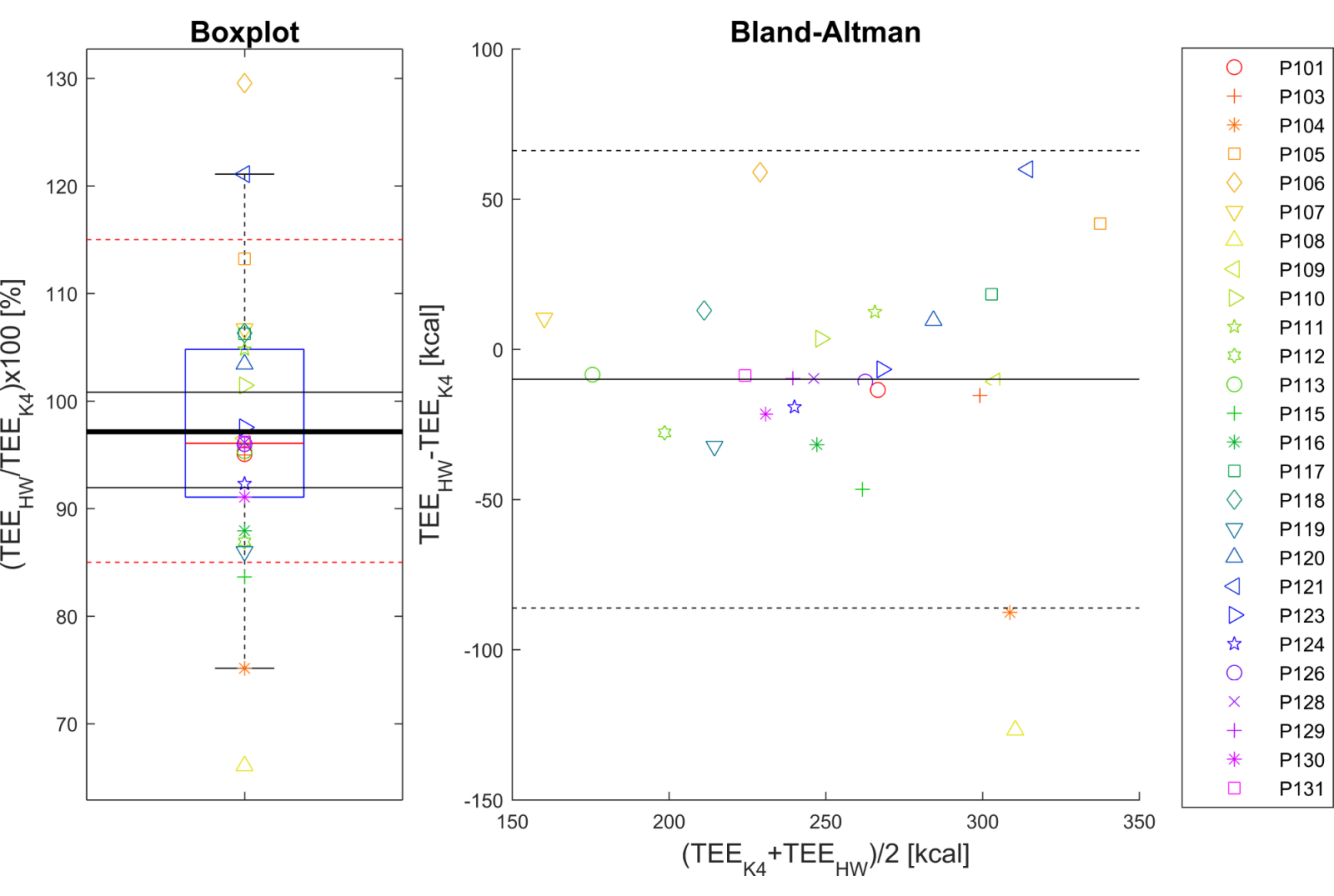
Boxplot (left-hand panel) of the ratio of cumulative total energy expenditure (TEE) between the Philips health watch (HW) and the K4b2 (K4) reference. The thick black line indicates the mean of the data, the red dashed lines the predefined ±15% equivalence interval, and the other thin black lines represent the calculated 95% CI of equivalence. Right-hand panel: Bland-Altman plot of the cumulative TEE for the HW and the reference. The solid black line indicates the average bias and the dashed black lines represent the 95% limits of agreement. Symbols represent participants’ individual data as indicated in the legend and are the same for both panels.

The TOST evaluation was applied at a significance level of .05, for equivalence margins of ±15%, leading to rejection of both null hypotheses [62] and therefore the conclusion that cumulative total energy expenditure as measured with the health watch and the K4b^2^ were equivalent. Results of the cumulative total energy expenditure comparison between the Philips health watch and the COSMED K4b^2^ ambulatory metabolic system are shown in Figure 2. As can be seen in Figure 2, the mean error (97% ratio, 95% CI 92%-101%) in total energy expenditure estimation (indicated by the thick black line) was well within the predefined 15% range of equivalence (indicated by the red dashed lines). There was a mean underestimation of 10.0 kcal, SD 38.9 kcal, which in relative terms was 2.9%, SD 13.1%, of the average reference value. The mean absolute error amounted to 27.5 kcal, SD 28.7 (MAPE 10.0%, SD 8.7%).

### Resting Heart Rate

**Figure 3:**
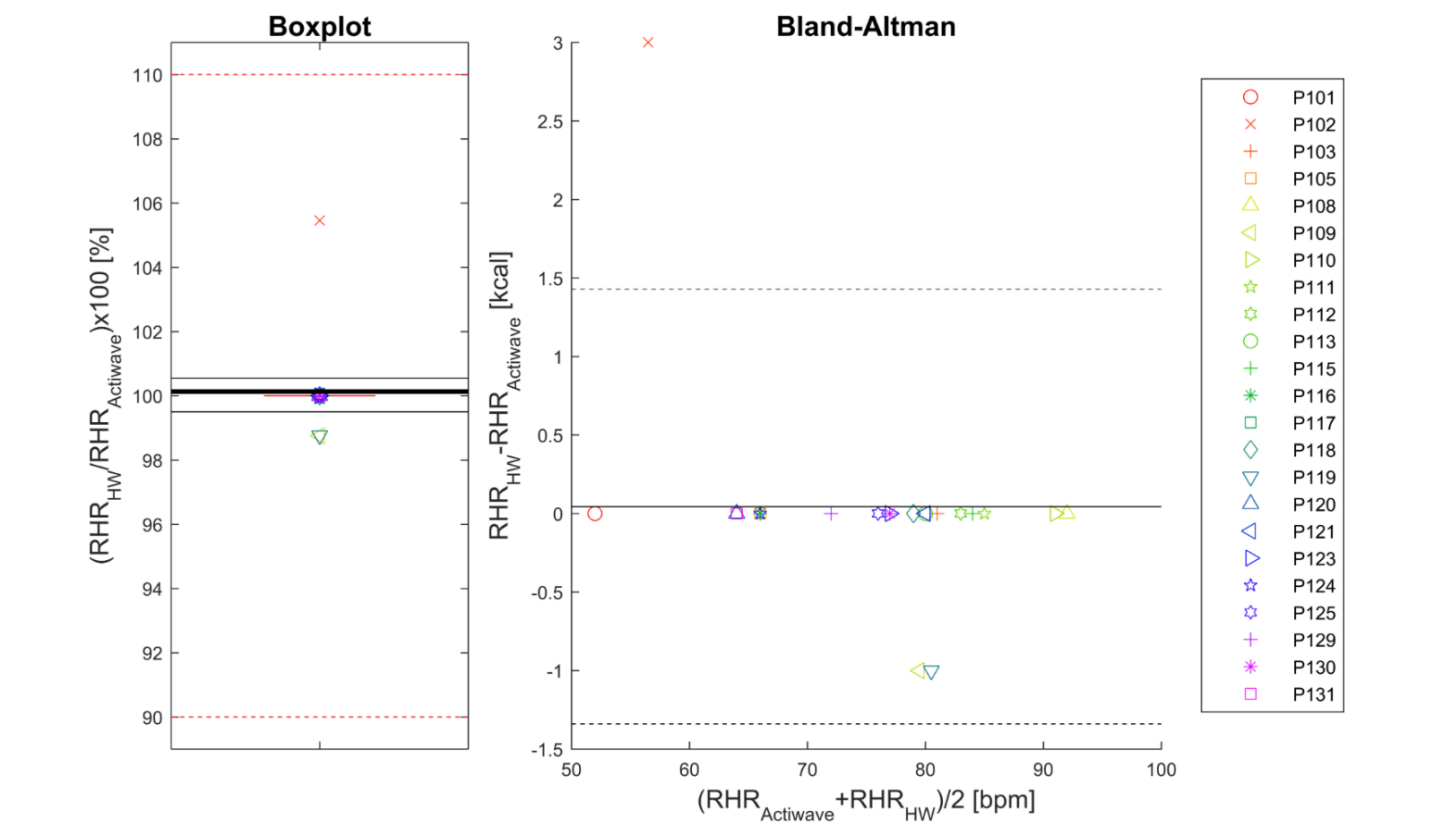
Boxplot (left-hand panel) of the ratio of resting heart rate (RHR) determined from the Philips health watch (HW) data during the rest protocol in the laboratory to the RHR determined from the Actiwave data during the rest protocol in the laboratory. The thick black line indicates the mean of the data, the red dashed lines the predefined equivalence interval, and the other black lines the calculated 95% CI of equivalence. Right-hand panel: Bland-Altman plot of the RHR from the HW and reference. The solid black line indicates the average bias and the dashed black lines represent the 95% limits of agreement. Symbols represent participants as indicated in the legend and are the same for both panels. bpm: beats per minute.

The TOST evaluation was applied at a significance level of .05, for equivalence margins of ±10%, leading to rejection of both null hypotheses [62] and therefore the conclusion that resting heart rate as derived from the health watch and resting heart rate as derived from the Actiwave ECG during the 15 minutes of rest in the laboratory test were equivalent. Results regarding the comparison are shown in Figure 3. The mean ratio was 100%, and the 95% CI was 99.5%-100.5%. In absolute terms, the mean absolute error was 0.2 bpm as most values were exactly equal to the Actiwave reference, with the maximum deviation being 3 bpm.

**Figure 4:**
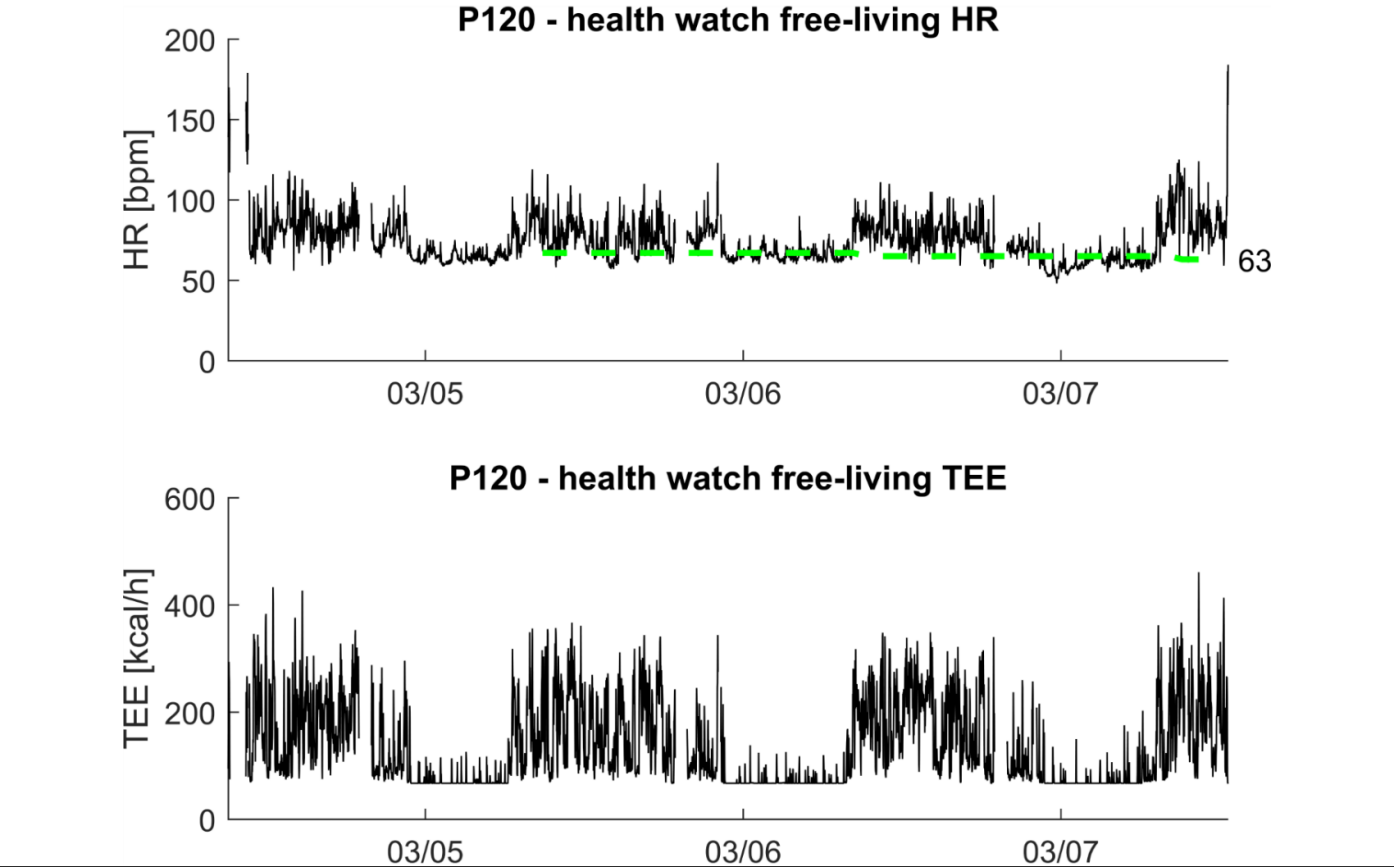
Example of free-living heart rate (HR), resting heart rate, and total energy expenditure (TEE) for participant P120. Top: Philips health watch HR as a function of time for the free-living portion of the trial (black) and the resting heart rate from the health watch (green dashed line; note that resting heart rate requires a 24-hour assessment and therefore day 1 does not have a resting heart rate value). Bottom: TEE as a function of time for the free-living portion of the trial. bpm: beats per minute.

Additionally, for each participant separately, we visually assessed whether the resting heart rate from the health watch coincided with the heart rate values at rest during the free-living part of the protocol. Figure 4 shows an example of a heart rate trace measured by the Philips health watch over 3 days of free-living conditions, together with the resting heart rate that was reported over time by the health watch (top), and the total energy expenditure estimation for the same time period (bottom). It can be seen that the resting heart rate corresponds with a low segment of the heart rate trace. In addition, the values sampled for each participant seem to correspond to time periods where subjects were awake and inactive, as can be deduced from the total energy expenditure graph, where sleep can be recognized as periods of low total energy expenditure with relatively low fluctuation.

### Heart Rate

Table 4 presents the results regarding heart rate measurement accuracy. The mean error was −1.7 bpm and the mean absolute error was 3.1 bpm. This corresponds to a mean percentage error of −1.3% and a MAPE of 3.1%. Of the available comparative data, the health watch measured heart rate within a difference of 10 bpm with the Actiwave ECG-based reference heart rate 94% of the time and within 10% of the ECG value 93% of the time (see Table 5).

**Table 4:** Heart rate errors compared with electrocardiogram-based reference that were calculated using 10-second nonoverlapping windows, as means and standard deviations, expressed in beats per minute and percentages.

Activity	Error, bpm^a^		Absolute error, bpm		Percentage error, %		Absolute percentage error, %	
	Mean	SD	Mean	SD	Mean	SD	Mean	SD
RHR^b^ protocol	0.6	1.8	1.5	1.7	1.0	3.1	2.2	3.0
Treadmill	−4.1	8.3	5.5	7.8	−3.7	7.6	5.2	7.1
Treadmill 5%	−0.3	4.2	3.9	4.2	−0.4	4.0	3.8	3.9
Ergometer bike	0.3	1.1	1.3	0.9	0.3	1.1	1.3	1.0
Cross trainer	−6.1	16.1	8.0	15.3	−4.6	11.9	6.1	11.2
Household	−2.3	5.7	5.7	4.3	−1.7	6.0	6.0	4.4
Desk work	0.9	1.8	1.7	1.7	1.4	3.1	2.4	2.9
Lying down	0.5	0.8	1.1	0.6	0.7	1.2	1.6	1.0
Standing	−0.3	1.4	2.0	1.4	−0.3	1.6	2.3	1.6
Walking	−4.3	9.2	7.1	7.8	−3.8	8.3	6.7	6.8
Cycling	−20.1	26.9	20.7	26.5	−14.5	18.4	15.1	18.0
Running	−6.7	9.7	8.3	8.7	−4.1	6.5	5.5	5.7
Total	−1.7	1.6	3.1	1.4	−1.3	1.5	3.1	1.4

^a^bpm: beats per minute.

^b^RHR: resting heart rate.

**Table 5:** Heart rate coverage parameters calculated using 10-second nonoverlapping windows for the individual activities as well as for the whole protocol.

Activity	Coverage within 10 bpm^a^, %	Coverage within 10%, %
RHR^b^ protocol	98.8	97.8
Treadmill	81.8	81.8
Treadmill 5%	85.2	85.9
Ergometer bike	94.9	94.9
Cross trainer	83.0	84.8
Household	85.4	83.8
Desk work	93.3	92.1
Lying down	90.8	90.3
Standing	93.2	92.9
Walking	64.5	70.7
Cycling	61.7	62.5
Running	72.2	88.7
Total	93.8	93.1

^a^bpm: beats per minute.

^b^RHR: resting heart rate.

### Step Counting

**Figure 5:**
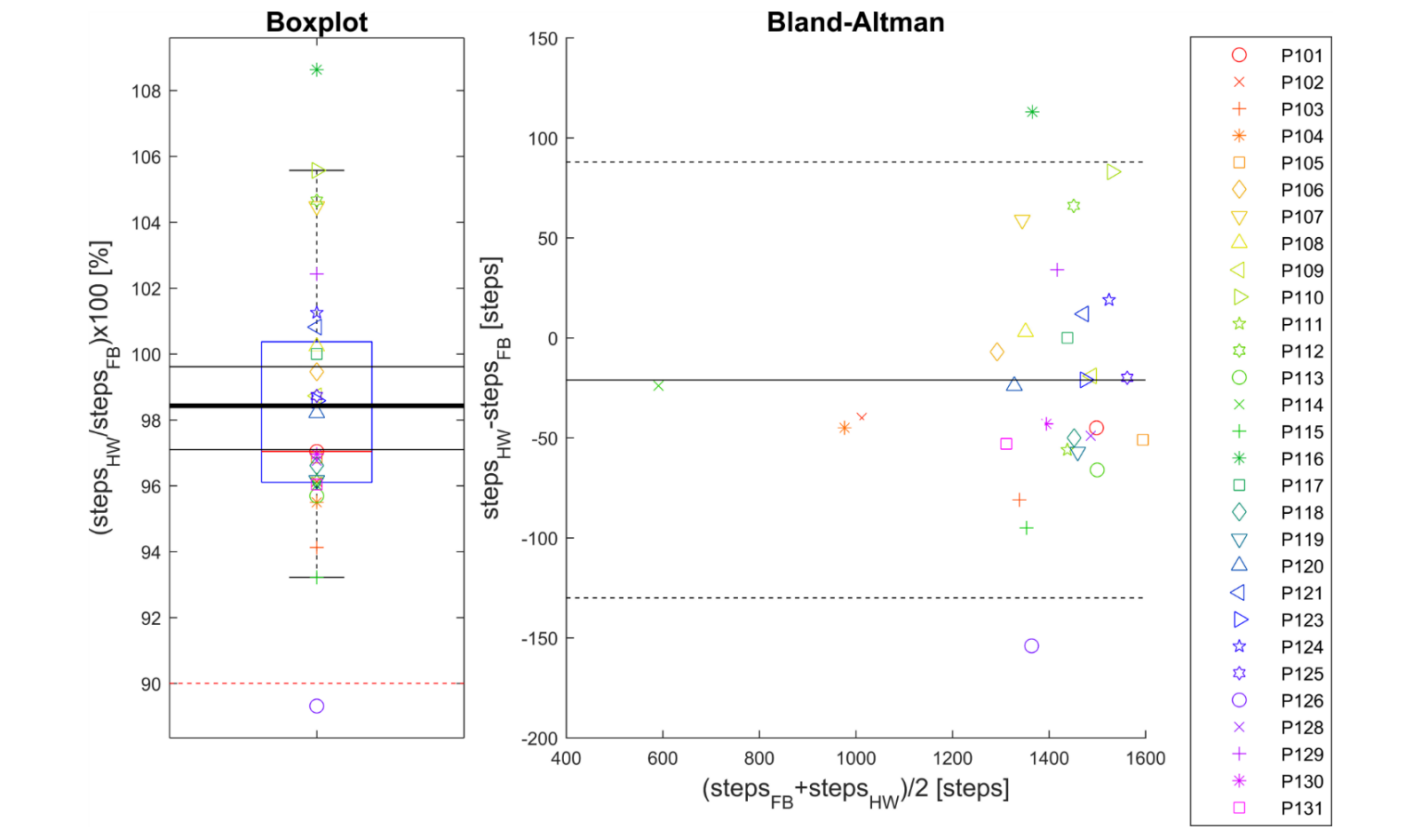
Boxplot (left-hand panel) of estimated step count as ratio between the health watch (HW) and the waist-worn reference (Fitbit One, FB), for all walk activities combined. The thick black line indicates the mean of the data, the red dashed lines the predefined equivalence interval, and the other black lines the calculated 95% CI of equivalence. Right-hand panel: Bland-Altman plot of the estimated step count of the HW and the reference measurement. The solid black line indicates the average bias and the dashed black lines represent the 95% limits of agreement. Symbols represent participants as indicated in the legend, and the legend is the same for both panels.

Compared with the step count reported by the waist-mounted reference, an overall (average) underestimation of 21 steps was observed corresponding to an overall error of −1.6% (see Table 6). Again, the TOST evaluation was applied at a significance level of .05, for equivalence margins of ±10%, leading to rejection of both null hypotheses and therefore the conclusion that cumulative steps as estimated by the health watch and by the waist-mounted counter were equivalent. The calculated 95% CI boundaries for all walking activities combined were at 97.1% and 99.6% (Figure 5).

**Table 6:** Errors in step count estimation when compared with the waist-mounted reference, based on the total number of steps for each activity.

Activity	Mean error, steps		Mean absolute error, steps		Mean percentage error, %		Mean absolute percentage error, %	
	Mean	SD	Mean	SD	Mean	SD	Mean	SD
Treadmill (n=27)	−13.0	11.5	13.4	11.1	−3.7	3.4	3.9	3.2
Treadmill 5% (n=29)	0.0	33.2	19.8	26.3	0.3	12.9	7.3	10.5
Walking (n=28)	−17.6	16.9	18.0	16.4	−6.5	11.0	6.6	10.9
Running (n=29)	8.3	42.2	26.9	33.2	2.4	10.8	6.4	9.0
All walk activities^a^ (n=29)	−21.1	55.6	47.9	34.3	−1.6	4.0	3.5	2.4

^a^The last row shows the observed error for the total number of steps of all walking activities.

### Activity Type Recognition

Overall, more than 90% of the time the correct activity type was identified by the Phillips health watch during the annotated laboratory activities (Table 7).

**Table 7:** A confusion matrix denoting the percentages of correct and incorrect activity type classifications compared with reference annotated activity types.

Annotated activity types	PHW^a^ activity type classification, %			
	Other	Walk	Run	Cycle
Other	99.3	0.5	0.0	0.2
Walk	5.5	94.2	0.1	0.3
Run	3.6	6.2	89.9	0.2
Cycle	8.1	0.4	0.0	91.6

^a^PHW: Philips health watch.

### Resting Respiration Rate

**Figure 6:**
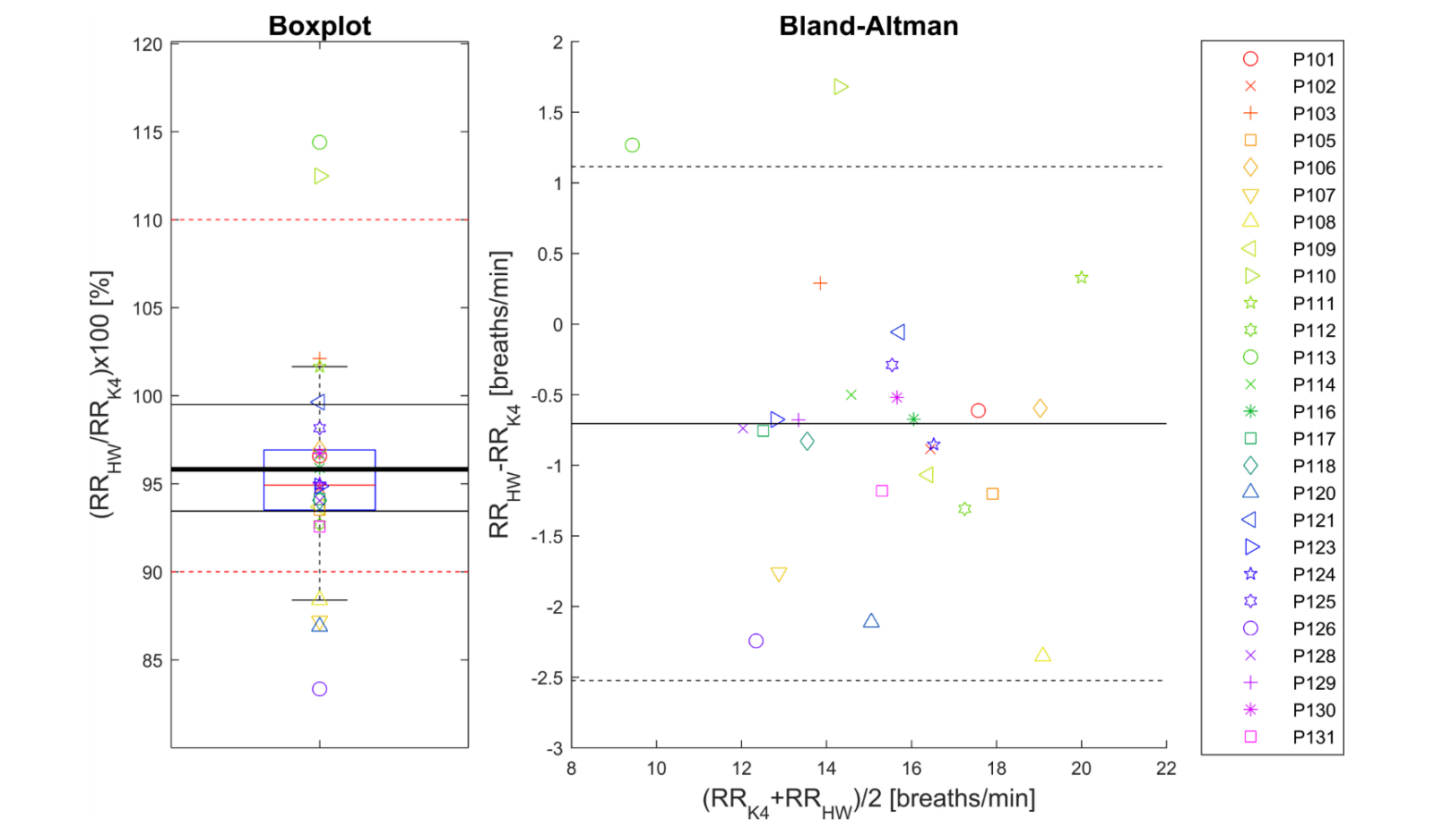
Boxplot (left-hand panel) of the ratio of estimated respiration rate (RR) from the health watch (HW) and the K4b2 (K4) reference, during rest. The thick black line indicates the mean of the data, the red dashed lines the predefined equivalence interval, and the other black lines the calculated 95% CI of equivalence. Right-hand panel: Bland-Altman plot of the estimated RR from the HW and from the reference measurement. The solid black line indicates the average bias and the dashed black lines represent the 95% limits of agreement. Symbols represent participants as indicated in the legend; the legend is the same for both panels.

The TOST evaluation indicated that respiration rate at rest as measured by the health watch and the K4b^2^ were equivalent (.05 significance level, ±10% equivalence margins). Figure 6 shows the boxplot of the ratio between the health watch and the K4b^2^ respiration rate, both averaged over the 15-minute rest period at the beginning of the laboratory protocol, with the calculated 95% CI boundaries of 93.4% and 99.5%. These data indicate a slight underestimation of respiration rate compared with the reference by -0.7, SD 1.1, breaths per minute. In percentages this amounted to an error of -3.8, SD 8.1, percent. The absolute mean error was 1.2, SD 1.0, breaths per minute (MAPE 8.3%, SD 7.0%).

## Discussion

### Principal Findings

In this study the measurement accuracy of the Philips health watch, a wrist-worn heart rate and activity monitor, was evaluated against (medical) reference instruments. Resting heart rate was determined on heart rate sampled over a 15-minute resting protocol in sitting position and fell within 3 bpm of the Actiwave ECG comparator. During a protocol covering a variety of activities of daily life, the health watch measured total energy expenditure on average within the predefined 15% accuracy compared with a K4b^2^ mobile metabolic system. These results indicate that the watch can provide valuable information that can help in the prevention and management of lifestyle-related chronic diseases by measuring and tracking resting heart rate and energy expenditure over time and interpreting these data in the context of a user’s personalized range of a parameter value or goal based on international standards (i.a. WHO, ACSM). The (automated) coaching that the companion app provides uses the information to offer further support to making a lifestyle change.

### Total Energy Expenditure

Energy expenditure estimation by means of wearable devices is not a new concept. There are many commercially available activity monitors that provide energy expenditure estimates; however, the reported accuracy of consumer-grade devices is highly variable [30,63-66]. Comparison across studies is hampered by differences in the type of reference measure (eg, doubly labeled water, metabolic chambers, or mobile metabolic systems) and differences in the type, intensity, and duration of activities performed during the validation (eg, standardized treadmill walking or free-living evaluation). Similar to our study, Lee et al [30] evaluated several consumer-grade physical activity monitors against a portable metabolic system (Oxycon Mobile) over a 69-minute standardized protocol of various activities. They reported MAPEs of 9.3% (BodyMedia FIT) up to 23.5% (Basis B1 band). Another study that resembled our design was performed by Bai and colleagues [67]. They evaluated activity monitors against an Oxycon Mobile metabolic system over an 80-minute standardized protocol. MAPE values ranged from 15.3% (BodyMedia Core) to 30.4% (Misfit Shine) in this study. Most recently, Nelson et al [68] evaluated several activity monitors over a 65-minute protocol covering 10 minutes of rest and a selection of 11 different activities using a COSMED K4b^2^ as reference. They reported MAPEs ranging from 13% to 35% for energy expenditure prediction over the different activities. In this study, the Philips health watch MAPE for total energy expenditure was 10%, which is highly accurate for this type of device when compared with the performance reported in the aforementioned studies. It is important to realize that the heart rate and acceleration measurements will give an estimation of total energy expenditure, which is less accurate than objective measurement techniques such as doubly labeled water or indirect calorimetry by means of ambulatory metabolic systems. However, these measurement methods are not feasible for long-term 24-hour monitoring of total energy expenditure in daily life and are not readily accessible to consumers [22]. In comparison with self-report questionnaires for physical activity, the Philips health watch provides a more objective measurement of total energy expenditure that is well suited for long-term, noninvasive monitoring. Similar to other validation studies of energy expenditure estimation, our study was limited to an evaluation of participants for a limited time frame at our test facility, as home testing was not practically feasible with regard to obtaining within-person reference measurements [63]. A strength of the study was that the protocol included activities of daily life, such as desk work, household activities, and activities performed outdoors, in addition to more traditional treadmill-based protocols. This will provide a better reflection of daily life performance [69].

### Resting Heart Rate

In this study the resting heart rate value determined with the health watch was found to be equivalent to that of the Actiwave reference (Figure 2). With the mean bias centered around 0 and a maximum deviation of 3 bpm, the health watch is suited for inspection of resting heart rate as a risk factor, as dose-response investigations often report increments in hazards for 5-10 bpm increments of resting heart rate [31,32,34,70]. A limitation of the study was that it was not possible to evaluate the resting heart rate produced by the health watch against a gold standard because there is currently no international consensus on a standardized manner to obtain resting heart rate values. We did, however, follow current recommendations by assessing resting heart rate based on multiple heart rate samples during a 15-minute resting protocol [50,55]. Furthermore, inspection of individual free-living heart rate traces indicated that the resting heart rate estimates of the health watch correspond to low heart rate levels during nonactive, although nonsleep, periods throughout the day. A strength of this method of obtaining resting heart rate by means of continuous heart rate monitoring is that it is much less influenced by circadian or temporary factors such as the “white coat effect,” which can confound the measurement [51,71].

### Heart Rate

Continuous heart rate logging of the Philips health watch was evaluated against Actiwave measurements over the complete duration of the protocol. Values sampled at 1 Hz were averaged over 10-second nonoverlapping windows. Parak and Korhonen [29] performed a similar comparison over a 50-minute protocol of various activities with a Mio Alpha, a wrist-worn device using a predecessor sensor module to that of the Philips health watch. They found a mean absolute error of 4.43 bpm and MAPE of 5.23% using 5-second nonoverlapping windows. In comparison, the Philips health watch performed better in this study with a mean absolute error of 3.1 bpm and MAPE of 3.1% (Table 4). Additionally, the coverage of the health watch within 10% of the reference device was higher in this study with 93.1% (Table 5) versus 87.5% reported in the study by Parak and Korhonen. For some activities, we observed lower accuracy and coverage presumably owing to the relative short duration of the activities. Our hypothesis was that the short duration of the activities resulted in relatively steep rising and dropping of heart rates, thus negatively affecting the estimation accuracy for only these activities compared with a protocol with longer activity durations resulting in more stable heart rates. Furthermore, during the months of testing (February and March) a temperature shift from the warmer indoor temperature to the lower outside temperature may have caused temporary localized vasoconstriction leading to lower coverage values for the outdoor activities walking and bicycling (Table 5). This phenomenon has been observed in an experimental setup by Maeda and colleagues [72], who demonstrated that the pulsatile AC (alternating current) component of the PPG signal is significantly lower at skin temperatures below 20°C compared with normal skin temperatures. This results in a significantly lower AC/DC (direct current) component ratio and reduces the correlation with ECG-based heart rate measurements. Although the accuracy of the health watch is equivalent to an ECG-based comparator for a high percentage of time, deviations due to, for example, a poor sensor-skin contact or movement artifacts are still possible. Attention to correct wearing of PPG-based devices is therefore important. Furthermore, for the purpose of this device, a 24/7 heart rate and activity monitor, a 93.1% coverage within 10% can provide a good overall representation of a user’s heart rate over the course of a day.

### Step Counting

Step counting of the health watch was compared against a waist-worn step counter over walking activities at different speeds, indoors on a treadmill and outdoors. From prior research, it is known that waist-worn devices generally have less error in step counts when compared with observed counts than wrist-worn devices [57-59,73]. Compared with the waist-worn counter, the health watch had a slight average overestimation of 0.3% for treadmill walking at 3 km/h at a 5% inclination and a small underestimation of −3.7% for treadmill walking at 4.5 km/h at 0% inclination (Table 6). These errors were smaller than those reported by Diaz et al [58], who found a mean underestimation of 16.3% and 10.6% when comparing a wrist-worn step counting device with observer counts over comparable slow and moderate speeds.

### Activity Type Recognition

Activity type recognition is useful for physical activity monitors as it can give insight to users into the duration of different types of activities that were performed over the course of a day and what amount of energy expenditure was associated with this. Furthermore, activity type classification provides the potential to enhance energy expenditure estimation [22,74-78]. Overall, more than 90% of the time the correct activity type was identified by the health watch during the annotated laboratory activities. This is a good result when comparing with other studies of automatic activity type recognition, where overall correct classifications range from 42% to 96% depending on the types of activities classified [79-82]. In Table 7 it can be seen that the least accurate activity type was running at 89.9%. Running was classified as walking approximately 6% of the time. One reason that the running recognition was least accurate could be the fact that 2 participants were actually walking during the running part of the protocol.

### Conclusions

This study showed that the health watch can estimate total energy expenditure with 85% accuracy during daily life activities and measure resting heart with ±3 bpm accuracy during rest compared with medical device reference instruments. In addition, the secondary outcome parameters, heart rate, step counts, resting respiration rate, and activity type classification, showed high levels of accuracy. On the basis of these results the health watch can serve its medical purpose of measuring resting heart rate and total energy expenditure over time in an unobtrusive manner, thereby providing valuable data for the prevention and management of lifestyle-related chronic diseases.

## Abbreviations

AC: alternating current
ACSM: American College of Sports Medicine
bpm: beats per minute
DC: direct current
ECG: electrocardiogram
ISO: International Organization for Standardization
MAPE: mean absolute percentage error
NCD: noncommunicable disease
PPG: photoplethysmography
TOST: two one-sided tests
WHO: World Health Organization
